# The association between adherence to the New Nordic Diet and diet quality

**DOI:** 10.3402/fnr.v60.31017

**Published:** 2016-06-01

**Authors:** Helga Birgit Bjørnarå, Nina Cecilie Øverby, Tonje Holte Stea, Monica Klungland Torstveit, Elisabet Rudjord Hillesund, Lene Frost Andersen, Sveinung Berntsen, Elling Bere

**Affiliations:** 1 Department of Public Health, Sport and Nutrition, Faculty of Health and Sport Sciences, University of Agder, Kristiansand, Norway; 2 Department of Nutrition, University of Oslo, Oslo, Norway

**Keywords:** New Nordic Diet, diet score, Norwegian food-based guidelines, dietary intake, nutrient intake

## Abstract

**Background:**

Previous studies have reported a positive association between scoring on healthy Nordic diet scales and the intake of healthy foods and nutrients, and also with higher intake of meat, sweets, cakes, and energy in general. These studies have used the same food frequency questionnaire (FFQ) responses for constructing the diet score as for calculating intakes of foods and nutrients. Thus, it is not clear whether the coexistence of healthy and less healthy dietary aspects among adherers to Nordic diets would occur even though separate methods were applied for exploring these relations.

**Objective:**

To assess the association between adherence to the New Nordic Diet (NND), derived from an FFQ, and diet quality, determined from two 24-h dietary recall interviews.

**Design:**

In total, 65 parents of toddlers in Southern Norway answered the NND FFQ and two 24-h dietary recall interviews. NND adherence was determined from the FFQ and categorized into low, medium, and high adherence. The two 24-h recalls provided data for the intake of specific foods and nutrients, selected on the basis of the Norwegian food-based guidelines as an indicator of a healthy diet. The Kruskal–Wallis test was used for assessing differences in food and nutrient intake across NND groups.

**Results:**

High NND adherence derived from FFQ was associated with a high intake of fruits (*p*=0.004) and fiber (*p*=0.02), and a low intake of meat (*p*=0.004) and margarines (*p*=0.05), derived from recalls. A larger proportion of high NND adherers (68%) complied with the national dietary recommendation targeting meat intake compared with low NND adherers (29%) (*p*=0.04).

**Conclusion:**

The present study showed that higher NND adherence measured with FFQ was associated with a higher intake of selected healthy foods and nutrients, measured with recalls. However, a higher intake of meat, sweets, and energy, as earlier reported, was not observed.

As a result of the demonstrated protective effects of the Mediterranean diet on disease (1–3) and mortality (2), the possible protective effects of other regional diets have gained attention. In the Nordic countries, dietary scores have been constructed in order to explore adherence to different aspects of the Nordic diets with expected health-promoting effects (4–6). Recently, observational studies have reported that compliance with Nordic diets is associated with lower mortality (4, 7) and a reduced risk of non-communicable diseases (5, 8–13). However, the evidence is not quite consistent, as other studies have failed to demonstrate associations between Nordic diets and breast cancer (14), colorectal cancer (15), or type 2 diabetes (16) and have reported equivocal associations with cardio-metabolic risk factors (17).

Three studies have examined dietary composition and nutrient intake related to three different Nordic diet scores, concluding that high scores were associated with an increased intake of healthy foods (5, 18) and essential nutrients (5, 6, 18). In a sample of Swedish women (18), higher scores on the Healthy Nordic Food Index (HNFI) were associated with a higher intake of the six food groups included in the score, that is, apples/pears, cabbage, root vegetables, whole grain bread, oatmeal, and fish/shellfish, in addition to fiber and higher micronutrient density. Likewise, participants in the Norwegian Mother and Child Cohort Study (MoBa), who attained higher ratings in the New Nordic Diet (NND) score, reported a higher consumption of healthy foods like whole grains, fish, fruits, and vegetables, and thus increased fiber intake and overall higher nutrient density (5). In a representative sample of the Finnish population, increased compliance with the Baltic Sea Diet Score implied a higher intake of fiber, iron, vitamin D, and folate, and a decreased intake of saturated fatty acids (SFAs) and alcohol (6). Moreover, high diet scores were associated with being more physically active (6, 18) and more likely to exercise (5).

Nevertheless, not all reported associations between diet scores and food intake have been in a healthier direction. In Norway, ‘high’ NND adherers were reported eating slightly more meat, cakes, and desserts than ‘low’ NND adherers (5), while Swedish women with high scores on the HNFI also reported a higher intake of less healthy foods such as processed meat and sweets (18). Moreover, in the Finnish sample higher intake of sodium and lower intake of polyunsaturated fatty acids (PUFAs) was observed among adherers to the Baltic Sea Diet Score (6). In all three studies, a high score was positively associated with energy intake (5, 6, 18).

These three studies, examining the association between adhering to Nordic diets and food/nutrient intake, all used the same food frequency questionnaire (FFQ) for constructing the diet score as for calculating intakes of foods and nutrients. Therefore, based on the previously reported coexistence of healthy and less healthy dietary aspects among adherers to predefined healthy Nordic diets, the aim of the current study was to assess the association between adherence to the NND score, derived from an FFQ, and diet quality, determined from two 24-h dietary recall interviews.

## Methods

### Design and study sample

The present data originate from the project Healthy and Sustainable Lifestyle, which in 2014 collected data in collaboration with the Child Food Courage project (19). As part of these projects, a web-based questionnaire was constructed to explore lifestyle behaviors, self-perceived health and quality of life, as well as basic demographic and socioeconomic variables (e.g. sex, age, height, weight, ethnicity, and educational attainment) among parents of toddlers. For the current methodological study, a convenience sample, consisting of parents of toddlers born between 2008 and 2011, was recruited through kindergartens. The leader of each kindergarten was asked to distribute the study invitation to eligible parents who were able to speak and read Norwegian. For each child, either the mother or the father could participate. Parents were informed about the purpose and implications of the study through a web page and via e-mail distribution. In total, 1,191 parents from 19 kindergartens in the county of Vest-Agder, Southern Norway, were invited to participate. A total of 86 (7%) parents signed up. Parents provided consent electronically, followed by administration of the questionnaire survey by e-mail. Subsequently, two 24-h dietary recalls were conducted by telephone 2–4 weeks apart, level of physical activity was recorded objectively for seven consecutive days, and anthropometric measurements were undertaken (height and body mass). Data collection was conducted between March and August 2014. In total, 56 parents (65% of those who signed up) completed all measurements, that is, the electronic questionnaire, two dietary recalls, and the physical activity assessment, while 65 parents (76%) completed the questionnaire and two dietary recalls, and 75 parents (87%) completed the questionnaire only.

## Measures

### The New Nordic Diet score

The electronic questionnaire incorporated an FFQ assessing participants’ habitual frequency of intake of selected foods, without specification of amounts consumed. The foods assessed included foods that are part of the concept of a NND, which has been suggested due to its inherent properties that potentially promote health, environmental sustainability, and food traditions (20), without compromising palatability (21). The NND consists of healthy foods native to the Nordic climate or foods that can be produced or cultivated in the Nordic climate, like certain fruits, berries, root vegetables, cabbages, whole grains, wild fish and game, potatoes, and rapeseed oil (20, 22). The NND score was previously developed to capture adherence to the NND in observational studies (5), and has recently shown acceptable test–retest reliability (23). The NND score comprises 10 subscales selected to summarize meal pattern and habitual intake of typical Nordic foods. Appendix 1 describes the components underlying the construction of the 10 subscales in the present study, including questionnaire items and frequency options. In the present study, the number of items forming the basis for each subscale ranged from 1 to 5, a total of 24 questions. Each subscale was dichotomized by the sex-specific median and assigned values of ‘0’ or ‘1’, with ‘1’ indicating a more frequent consumption of main meals (subscale 1) or a more favorable intake of selected foods (subscale 2–10). Each subscale was assigned equal weightage, and adding the subscales yielded a score ranging from 0 to 10, with increasing scores indicating higher compliance with the NND. The total score was trichotomized grouping participants into ‘low’ (0–3 points), ‘medium’ (4–5 points), and ‘high’ (6–10 points) adherence to the NND (5), with cutoffs for groupings determined to obtain the most equally sized groups.

### 24-Hour dietary recall interviews

Two unannounced 24-h dietary recalls were collected by telephone, 2–4 weeks apart by two trained interviewers, after completion of the FFQ. Each interview lasted for approximately 20–30 min, aiming to obtain detailed information on all foods and beverages consumed by the participants in the period between waking up on the preceding day and waking up on the interview day. A booklet containing photographs of various portion sizes for common foods and standard sizes of glasses, cups, and plates (24) was available on the project's web page to ease the estimation of portion sizes from memory. Dietary intake was reported for one weekday and one weekend day by 21 participants (32%), of whom 18 participants (86%) reported for a Sunday, while 3 participants (14%) reported for a Saturday. The remaining 44 participants (68%) reported dietary intake for two weekdays, due to feasibility. Dietary information was converted into daily energy and nutrient intakes using the food calculation software KBS V 7.0, linked directly to the food composition database N3. The Norwegian food composition table from 2006 (25) forms the basis for this food composition database, which is also supplemented with additional food items from reliable sources. The 24-h recall functionality of the KBS program was developed specifically for the Norkost 3 study, which represents the latest national dietary survey conducted among a representative sample of Norwegian adults (24). Nutritional supplements were excluded from the calculations, as food intake per se was that of interest in this study, and what corresponds with the concept NND.

In order to assess diet quality across NND adherence, specific foods and nutrients assessed by the two 24-h dietary recalls were selected, based on the official Norwegian food-based guidelines (26) as an indicator of a healthy diet. Foods assessed were ‘Vegetables (fresh and frozen)’, ‘Fruits and berries (fresh)’, ‘Fruit juice’, ‘Whole grain products’, ‘Refined grain products’, ‘Fish’, ‘Meat’, ‘Low fat dairy products’, ‘Fatty dairy products’, ‘Vegetable oils’, ‘Margarines’, ‘Butter’, ‘Chocolate, candies and sugar-sweetened beverages’, and ‘Water’. Selected nutrients were fiber, added sugar, and sodium. In addition, we assessed energy intake across NND groups. Also, the proportion from each NND adherence category meeting the following quantitative Norwegian food recommendations was calculated; ‘Eat at least five portions of vegetables, fruits, and berries every day’, ‘Eat whole grains every day’, ‘Eat fish for dinner two to three times a week and preferably also as sandwich spread’, ‘Choose lean meat and lean meat products. Limit the amount of processed meat and red meat’, ‘Choose foods containing little salt, and limit the use of salt for cooking’, and ‘Avoid sugar rich foods and beverages for everyday use’. Calculations were performed in line with the methodology of the Norkost 3 study (24), entailing that for whole meal bread, 40% of the product weight was accounted for as whole grains, while for muesli/mixed cereals, 50% of the product weight was included. Further, cut-offs for compliance were set at 70 g whole grain/day for women and 90 g/day for men. Recommendations regarding fish intake and meat consumption were operationalized into daily intake, as recommended weekly amounts are 300–450 g of fish (ready to eat), and <500 g of red and processed meat (ready to eat), for both females and males. Consequently, due to the features of the food calculation software used (KBS V 7.0), 40% of the product weight of processed fish products was included (24), and for meat intake the recommended commodity weight (750 g/week) (27) represented the cut-off.

Moreover, the habitual frequency of consumption of selected foods (i.e. vegetables, fruits and berries, fruit juice, whole grain products, refined grain products, fish, meat, and sweet pastries, candies, and sugar-sweetened beverages) across NND adherence groups was assessed using FFQ data. Although amounts were not specified, frequencies would allow for an examination of tendencies across groups, using the same FFQ data for determining NND adherence as for assessing dietary intake, in line with the methodology applied in the earlier studies (5, 6, 18).

### Physical activity and anthropometric measurements

To enable exploration of the physical activity level in the present sample, as one relevant sample characteristic, and also the relation between energy intake and energy expenditure, the monitor SenseWear Armband Mini (SWA; BodyMedia, Pittsburgh, Pennsylvania, USA) was used. SWA includes a 3-axis accelerometer, a heat-flux sensor, a skin temperature sensor, and a near-body ambient temperature sensor (28). Data from these sensors were combined with sex, age, body weight, and height to estimate physical activity intensity and energy expenditure using algorithms developed by the manufacturer. Participants were instructed to wear the monitor on the upper left arm (on the triceps, at mid humerus point) for seven consecutive days, only removing it for bathing, or any other water activity. Those with a nickel allergy were discouraged from participating (*n*=5), as wearing the monitor may cause skin rashes due to 8% nickel content. Data were downloaded using SenseWear Professional V.8.1 (BodyMedia, Pittsburgh, Pennsylvania, USA). A valid day was defined as at least 80% (19.2 h) wearing time, and a minimum of four valid days with at least one weekend day was required for participants to be included in the analyses (29, 30). Data were calculated and reported as mean values per day. Participants exceeding 21.5 min/day with moderate and vigorous physical activity, in bouts of at least 10 min duration, were classified as meeting the recommendations for physical activity (26, 31). The cut-off defining moderate to vigorous intensity was 3 metabolic equivalents (METs) (32). Anthropometric measurements were obtained by trained staff, with subjects barefoot and dressed in light clothes. Height was measured using a portable stadiometer with the head in the Frankfort plane, two measurements were taken and added with a third if the first two differed by >1%. The mean of the closest two measurements was calculated. Body mass was measured by a segmental multi-frequency bioelectrical impedance analysis (BIA), conducted with In Body 720 (Biospace Co., Ltd., Seoul, Korea). Body mass index (BMI) was computed, as this represents one significant and commonly included sample characteristic, and participants with a BMI≥25 kg/m^2^ were categorized as overweight/obese (33). In compliance with the measurement protocol, participants were instructed to abstain from exercise and food within 2 h of testing, and immediately prior to the measurement to avoid showering and sauna, and to empty their bladder. Pregnant women were excluded from the body composition measurements (*n*=1).

### Statistical analysis

Statistical analyses were performed with the statistical software package IBM SPSS Statistics version 22.0 (IBM Corp., Somers, New York, USA). To explore differences in sample characteristics across NND adherence categories, Chi-square test for independence (χ^2^) was used. Food consumption variables were skewed, thus the Kruskal–Wallis test was applied for assessing differences in food, nutrient and energy intakes, and energy expenditure, across NND categories. Results are presented as median and quartiles. Differences in compliance with the Norwegian quantitative food-based dietary guidelines according to NND adherence group was assessed with Chi-square. A two-sided *p*-value of <0.05 was considered statistically significant.

## Results

A total of 65 participants were included in the final analyses. Mean age in the study sample was 35.2 years (SD±5.0 years), 55 participants (85%) were females, 58 participants (89%) were native Norwegians, and 37 participants (57%) reported four or more years of university or college education (Table 1). Furthermore, 13 participants (20%) were overweight or obese, while 46 participants (82%) met the national recommendations on physical activity (26). No significant differences were observed in sample characteristics across NND categories (Table 1). Participants were categorized according to the NND score into low (*n*=17), medium (*n*=23), or high (*n*=25) NND adherence, representing 26, 35, and 39% of the sample, respectively. Among the 21 participants (32%) reporting dietary intake for one weekday and one weekend day, distribution across NND adherence groups was: low (*n*=5), medium (*n*=6), and high (*n*=10), representing 24, 28, and 48%, respectively.

**Table 1:** Selected characteristics of the study sample in total (*n*=65), according to NND adherence

	Degree of NND adherence				
		
	Whole sample (*n*=65)	Low (*n*=17)	Medium (*n*=23)	High (*n*=25)	
	*n* (%)	*n* (%)	*n* (%)	*n* (%)	p*
Sex					
Female	55 (85)	14 (82)	20 (87)	21 (84)	0.92
Age (yrs)					
20–34	31 (48)	8 (47)	12 (52)	11 (44)	
≥35–47	34 (52)	9 (53)	11 (48)	14 (56)	0.85
Ethnicity					
Native Norwegian†	58 (89)	16 (94)	21 (91)	21 (84)	0.54
Educational attainment					
Higher education‡	37 (57)	13 (77)	9 (39)	15 (60)	0.06
Weight status					
Overweight/obese§	13 (20)	4 (24)	5 (22)	4 (16)	0.81
Physical activity level					
Physically active||	46¶ (82)	11 (73)	16 (80)	19 (91)	0.40

NND, New Nordic Diet.

* *p*-values calculated from Chi-square test for independence (χ^2^).

† Both parents born in Norway.

‡ ≥4 years of university or college education.

§ Body mass index ≥25 kg/m^2^.

|| >21.5 min/day with moderate and vigorous physical activity, in bouts of at least 10 min duration, measured by the activity monitor SenseWear Armband Mini.

¶ For physical activity level *n*=56; 15, 20, and 21 parents categorized into low, medium, and high NND, respectively.

Different consumption of selected foods (Table 2) across NND groups was detected for meat (*p*=0.004), fruits and berries (*p*=0.004), and margarines (*p*=0.05), entailing that those classified as ‘low’ NND adherers reported the highest consumption of meat and margarines, while ‘high’ NND adherers reported the largest intake of fruits and berries. For the other foods assessed, that is, fresh and frozen vegetables, fruit juice, whole grain products, refined grain products, fish, low-fat dairy products, fatty dairy products, vegetable oils, butter, chocolate, candies and sugar-sweetened beverages, and water, no significant differences were observed. The relative intake of dietary fiber (E%) differed significantly across NND groups; fiber contributed with 2.7 E%, 2.4 E%, and 2.1 E% (*p*=0.02), in ‘high’, ‘medium’, and ‘low’ NND adherers, respectively. For added sugar and sodium, no differences according to NND classifications were found. Likewise, energy intake and energy expenditure did not differ across NND groups (Table 2).

**Table 2:** Daily energy expenditure, energy intake, and consumption of fiber, added sugar, sodium (Na), and selected foods, according to NND adherence

	Degree of NND adherence						
		
	Low (*n=*17)		Medium (*n*=23)		High (*n*=25)		
				
	Median†	(Q1, Q3)	Median	(Q1, Q3)	Median	(Q1, Q3)	p*
Energy expenditure (kJ)‡	11,026	(10,041, 12,203)	10,621	(10,040, 11,870)	11,456	(10,074, 12,436)	0.65
Energy intake (kJ)	9,361	(7,762, 12,200)	8,308	(7,418, 10,992)	8,883	(7,225, 10,961)	0.46
Fiber (g)	24.5	(21.8, 27.7)	26.4	(22.0, 32.0)	30.2	(23.0, 40.3)	0.07
Fiber (E%)	2.1	(1.6, 2.6)	2.4	(2.1, 2.7)	2.7	(2.2, 3.1)	0.02
Added sugar (g)	38.1	(17.8, 60.4)	27.8	(10.7, 40.4)	21.1	(12.7, 36.2)	0.19
Added sugar (E%)	6.9	(3.4, 8.8)	4.7	(2.5, 7.5)	4.4	(2.7, 6.7)	0.17
Na (mg)	2738.0	(2214.5, 3795.0)	2786.0	(1828.0, 3860.0)	2789.0	(1984.5, 3778.5)	0.92
Vegetables (fresh and frozen)	139.9	(86.8, 218.6)	140.6	(76.0, 198.5)	164.4	(110.6, 243.5)	0.56
Fruits and berries (fresh)	102.5	(60.8, 207.8)	150.0	(120.5, 305.0)	267.5	(187.6, 348.8)	0.004
Fruit juice	93.8	(0.0, 312.5)	0.0	(0.0, 187.5)	0.0	(0.0, 122.5)	0.15
Whole grain products	57.7	(33.6, 110.0)	53.7	(30.1, 117.6)	67.2	(34.0, 136.0)	0.60
Refined grain products	130.2	(52.7, 169.7)	86.2	(51.3, 166.0)	74.9	(38.7, 162.7)	0.67
Fish||	21.3	(0.0, 60.1)	5.0	(0.0, 85.3)	38.5	(0.0, 86.8)	0.53
Meat¶	167.4	(134.6, 233.8)	116.5	(54.2, 189.6)	102.0	(60.8, 128.8)	0.004
Low fat dairy products	110.0	(12.0, 283.15)	142.5	(62.5, 320.3)	125.0	(49.8, 406.3)	0.60
Fatty dairy products	146.0	(71.7, 187.3)	75.2	(40.0, 154.9)	88.0	(57.0, 155.4)	0.20
Vegetable oils	1.1	(0.0, 5.1)	0.0	(0.0, 3.0)	0.3	(0.0, 2.3)	0.63
Margarines	7.5	(0.7, 19.6)	5.5	(2.5, 11.6)	1.1	(0.0, 7.9)	0.05
Butter	5.6	(3.2, 12.1)	5.0	(1.6, 10.2)	6.8	(2.2, 16.7)	0.50
Chocolate, candies, and sugar-sweetened beverages	42.0	(6.3, 205.0)	24.5	(1.0, 163.1)	4.0	(0.0, 64.5)	0.15
Water	1000.0	(601.9, 1297.0)	1215.0	(812.5, 1893.8)	1120.0	(795.0, 1452.5)	0.33

NND, New Nordic Diet.

* Kruskal–Wallis test was used to derive *p*-values.

† Median and quartiles were calculated from two 24-h dietary recalls.

‡ For energy expenditure (measured by the activity monitor SenseWear Armband Mini) and energy intake (assessed by two 24-h dietary recalls). *n*=56; 15, 20, and 21 parents categorized into low, medium, and high NND, respectively.

|| Includes lean fish, fatty fish, fish products, and selected fish toppings.

¶ Includes poultry, pork, beef, game (all unprocessed), ground meat, and processed meat (salted meat, minced meat, sandwich meat, and liver paste).

Regarding the frequency of habitual food intake (results not shown) measured with FFQ, significant differences across NND adherence groups were found for all foods except from fruit juice. ‘High’ NND reported to eat vegetables, fruits and berries, whole grain products, and fish more frequently than ‘low’ NND, while ‘low’ NND recorded more frequent consumption of refined grain products, meat, and sweet pastries, candies, and sugar-sweetened beverages than ‘high’ NND.

Table 3 shows that a greater proportion of ‘high’ NND adherers complied with the guideline to ‘Choose lean meat and lean meat products and limit the amount of processed meat and read meat’, than ‘low’ NND adherers (68% versus 29%, *p*=0.04). For the remaining five recommendations of interest, no significant differences between NND adherence groups were found.

**Table 3:** Proportions meeting the quantitative recommendations incorporated in the official Norwegian food-based dietary guidelines, according to NND adherence

		Degree of NND adherence			
			
		Low (*n*=17)	Medium (*n*=23)	High (*n*=25)	
			
Quantitative dietary recommendations	Behavior required for scoring	% adhering to recommendation	% adhering to recommendation	% adhering to recommendation	p*
3: ‘Eat at least five portions of vegetables, fruits, and berries every day’†	>500 g/day	29	35	48	0.43
4: ‘Eat whole grains every day’	>70 g/day (women) >90 g/day (men)	35	35	40	0.92
5: ‘Eat fish for dinner two to three times a week and preferably also as toppings’‡	>43 g/day	29	39	48	0.48
6: ‘Choose lean meat and lean meat products. Limit the amount of processed meat and read meat§’	<107 g/day	29	61	68	0.04
9: ‘Choose foods containing little salt, and limit the use of salt for cooking’.	<6 g salt (NaCl) per day	24	39	36	0.56
10: ‘Avoid sugar rich foods and beverages for everyday use’.	<10 E% sugar/day	88	87	96	0.51

NND, New Nordic Diet.

* Proportions were calculated using chi-square.

† For those with an average intake of at least 100 g of fruit juice, 100 g of juice were included.

‡ Includes lean fish, fatty fish, fish products, and selected fish toppings.

§ Includes lean, red meat (unprocessed), ground meat, and processed meat (salted meat, minced meat, sandwich meat, and liver paste).

## Discussion

In the present study, the association between adherence to the NND, derived from an FFQ, and diet quality, determined from two 24-h dietary recall interviews, was assessed. In line with former findings, the trend was that ‘high’ NND adherers reported a more favorable diet in general (5, 6, 18), and a higher intake of fruits (5, 18) and dietary fiber (5, 6, 18). Contrasting previous findings (5, 18), neither higher intake of meat or sweets, nor higher energy intake or higher physical activity levels was observed among ‘high’ NND adherers (5, 6, 18).

The previously observed coexistence of healthy and less healthy dietary elements among adherers to predefined healthy Nordic diets could have different explanations. First, it may be real, that is, that those who achieve high scoring on the Nordic scales have higher intakes of a wide variety of foods, which may be characterized as both healthy and less healthy. High intake of healthy foods and beverages will most likely have positive health effects, in spite of unhealthy elements in the diet. This aspect may partly explain previous results, especially as higher scoring on the Nordic scales was associated with being more physically active, or more likely to exercise, as well. Dietary factors not included when constructing a specific diet score could confound true associations between the healthier aspects of the diet and relevant outcomes. An example is meat, which has been reported to associate positively with colorectal cancer (34), and also with compliance to the HNFI (18). These relations may partly explain the lack of an inverse association between greater scoring on the HNFI, and colorectal cancer (15). Second, the scales assessing adherence to the different Nordic diets might be biased as a result of *the consistency motif* (35), that is, participants falling into a pattern of similar responses when answering comparable questions, which is a tendency that could apply to FFQs. Those reporting to eat more of the healthy Nordic foods might also erroneously report eating more of certain less healthy foods. If so, it may be debatable whether the dietary scores actually capture what they intend to capture. Third, artificial covariance (35) could have biased the earlier reported associations, due to using the same questionnaire responses for deriving the dietary score and for calculating food and nutrient intakes. In turn, such false associations could result in invalid inferences regarding diet-health relations. Since measurement errors would be less correlated if applying separate methods for these two operations (36), it may be favorable to construct the diet score from an FFQ, while using dietary recalls for estimating intake of foods and nutrients.

The FFQ was unfortunately not tested for validity, and misreporting, especially the underreporting of foods generally considered unhealthy, is a common challenge when data are self-reported (36, 37). However, if randomly distributed, misreporting should still allow the ranking of participants into groups according to intake. Besides, although the underlying concept of interest was NND adherence at the group level, the limited sample size in this study ideally calls for more than two 24-h recall interviews to reduce the influence of day-to-day variations in food consumption. This uncertainty seems to be reflected through the lack of a consistent trend in the results, and especially foods eaten more seldom, like fish, are the most sensitive for day-to-day variations. Moreover, all groups reported considerably lower energy intake than the objectively measured energy expenditure. Possible explanations might be increased activity levels caused by awareness of being observed (38), low energy intake as a result of the misreporting or underreporting of food consumption, or poor repeatability (37), due to the wide variations in food intake from one day to another.

On the other hand, our findings concerning the frequency of habitual food consumption, determined from the FFQ, revealed the same trend as when using separate methods. Hence, although frequencies are not the same as amounts, different observations in the current study compared with the earlier studies on Nordic scales might be related to sample characteristics. A homogenous and selective sample in the present study, in addition to recently collected data, could possibly imply a sample following a healthier diet than parents of toddlers in general, and therefore reduced generalizability. And, since dietary patterns are likely to change over time, the Nordic scales may capture other dietary aspects today, than when exploring data collected 10–20 years ago. In other words, the present results might indicate that the NND score, and similar Nordic scores, capture healthy diets to a larger degree when applied in more contemporary samples.

The results of the current study should be interpreted in the context of several limitations. As mentioned above, the study sample was selective and homogenous, that is, the majority being females, native Norwegians, and highly educated, probably caused by a very low response rate (7%). Together with a small sample size, these characteristics restricted study power, eligible statistical analyses (e.g. sub-group analyses), and generalizability to the population in general. A notably larger amount of the present sample complied with the recommended physical activity level (26), and fewer were overweight or obese, compared with a representative sample of the Norwegian adult population (39), that is, 82% versus 32%, and 20% versus 48%, respectively. Participating parents might have been more health-conscious and more likely to adhere to a favorable lifestyle, including a healthier diet. Unlike the former studies exploring the HNFI (18), the NND (5), and the Baltic Sea Diet Score (6), differences across NND adherence categories were not detected concerning age, educational level, BMI, physical activity level, or energy intake, expressing the homogeneity of the sample. Yet, lack of differences could also be a result of the limited sample size.

Regarding dietary scores as a method for quantifying adherence to dietary patterns, subjectivity is introduced related to the selection and scoring of included components, cut-off points, and so on (40, 41). Importantly, although reflecting a larger part of the overall diet, diet scores do not cover all aspects of diet, meaning that other food items not incorporated into the scale could bias the associations under investigation. Also, as cut-offs for the NND score were determined by the median, dietary behavior required for scoring is sample specific, and caution must be exercised when generalizing the results. In light of the sample characteristics, it is plausible that the diet underlying ‘low’, ‘medium’, or ‘high’ NND adherence entailed higher diet quality in the present sample compared to a more representative sample. Still, this procedure for determining cut-offs is in line with the methods applied in previous studies exploring relations between predefined dietary patterns and various health parameters (4–6, 42).

In addition to the use of separate methods for determining NND adherence and calculating intakes of foods and nutrients, it may be a study strength that the questionnaire was recently developed, and thus provided contemporary data. Previous studies derived dietary scores from data collected between years 1991 and 1999 (18), 2002 and 2008 (5), and in 2007 (6), implying that dietary patterns might have changed. On the other hand, it could be a disadvantage if NND adherers in the current study were familiar with the proposed favorable characteristics of the foods included in the NND, and gave the anticipated most desirable answers to the questions. Moreover, repeated 24-h recall interviewing is considered one appropriate method for collecting representative dietary data at group level, entailing less participant burden than dietary records (36, 43).

Unequal methodological approaches, or a selective and more recent sample, might partly explain discrepancies in the present findings as compared with earlier studies (5, 6, 18), that is, the previously observed associations between adherence to healthy Nordic diets and the intake of less healthy foods not part of the diets under investigation (21, 22). Nevertheless, considering the limitations of the current study, these associations should be further explored in larger and more heterogeneous samples in order to draw conclusions. Also, when applied in epidemiological studies, potential confounding lifestyle and dietary factors not included in the dietary score should be accounted for, since residual confounding could distort explored associations. Increased knowledge concerning potential methodological bias, as discussed in the present study, would be of importance, due to the fact that such bias may result in false inferences regarding diet-disease relations.

## Conclusions

The present study assessed the association between adherence to the NND measured with FFQ and diet quality measured with two 24-h dietary recall interviews, and showed that higher NND adherence was associated with a higher intake of selected healthy foods and nutrients. However, a higher intake of meat, sweets, and energy in general, as earlier reported in adherers to predefined healthy Nordic diets, was not observed, whether assessed by FFQ or 24-h dietary recall. Nonetheless, the methodological limitations in the current study imply replications in larger and more representative samples before inferences can be drawn regarding explanations for these partly differing results.

## Appendix

**Appendix 1:** The components underlying the construction of the 10 subscales within the NND score (*n*=75)

Subscale	Related question(s)	Response alternatives and coding	Calculations (min-max)	Median=cut-off	Dietary behavior associated with scoring
Meal pattern	How often do you eat -Breakfast -Lunch -Dinner -Evening meal/supper	Never=0 Less than once a week=0.5 Once a week=1 Twice a week=2 Three times a week=3 Four times a week=4 Five times a week=5 Six times a week=6 Every day=7	Sum of answers to the four questions (0–28)	Women: 25.0 Men: 25.0	Women: <25.0=0 ≥25.0=1 Men: ≤25.0=0 >25.0=1
Nordic fruits	How often do you eat typical Nordic fruits (apple, pear, plum)	Never=0 Less than once a week=0.5 Once a week=1 Twice a week=2 Three times a week=3 Four times a week=4 Five times a week=5 Six times a week=6 Every day=7 Several times a day=10	No calculation (0–10)	Women: 4.0 Men: 3.5	Women: ≤4.0=0 >4.0=1 Men: ≤3.5=0 >3.5=1
Root vegetables	How often do you eat root vegetables (e.g. carrot, rutabaga, onion)?	Never=0 up to Several times a day=10	No calculation (0–10)	Women: 5.0 Men: 4.5	Women: ≤5.0=0 >5.0=1 Men: ≤4.5=0 >4.5=1
Cabbages	How often do you eat cabbages (e.g. cauliflower, broccoli, Brussels sprouts, kale)?	Never=0 up to Several times a day=10	No calculation (0–10)	Women: 3.0 Men: 3.0	Women: ≤3.0=0 >3.0=1 Men: <3.0=0 ≥3.0=1
Potatoes vs. rice/pasta	How often do you eat -Potatoes -Rice -Pasta	Never=0 up to Several times a day=10	Frequency of eating potatoes relative to eating rice and pasta combined: potatoes/(0.1 +rice+pasta) (0–100)	Women: 0.49 Men: 0.39	Women: ≤0.49=0 >0.49=1 Men: <0.39=0 ≥0.39=1
Whole grain breads vs. white breads	How often do you eat -Refined breads/bread rolls -Whole grain breads -Whole grain hard breads	Never=0 up to Several times a day=10	Frequency of eating whole grain breads and whole grain hard breads combined relative to eating refined breads: (whole grain breads+whole grain hard breads)/(0.1+refined breads) (0–200)	Women: 15.0 Men: 9.6	Women: ≤15.0=0 >15.0=1 Men: ≤9.6=0 >9.6=1
Oatmeal	How often do you eat oatmeal?	Never=0 up to Several times a day=10	No calculation (0–10)	Women: 1.0 Men: 0.75	Women: <1.0=0 ≥1.0=1 Men: ≤0.75=0 >0.75=1
Foods from the wild countryside	How often do you eat -Game (e.g. moose, reindeer, deer) -Lean fish (e.g. cod, Pollock, haddock) -Fatty fish (e.g. mackerel, herring, halibut) -Other seafood (e.g. shrimps, crabs, mussels -Berries	Never=0 up to Several times a day=10	Sum of answers to the five questions (0–50)	Women: 4.5 Men: 4.5	Women: <4.5=0 ≥4.5=1 Men: ≤4.5=0 >4.5=1
Milk vs. juice	How often do you drink -Milk -Fruit juice without added sugar	Never=0 up to Several times a day=10	Frequency of drinking milk relative to drinking fruit juice: milk/(0.1+juice) (0–100)	Women: 1.29 Men: 2.5	Women: ≤1.29=0 >1.29=1 Men: ≤2.5=0 >2.5=1
Water vs. sugar/artificially sweetened beverages	How often do you drink -Water -Sugar-sweetened beverages -Artificially sweetened beverages	Never=0 up to Several times a day=10	Frequency of drinking water relative to drinking sugar-sweetened beverages and artificially sweetened beverages combined: water/(0.1+sugar-sweetened beverages+artificially sweetened beverages) (0–100)	Women: 6.25 Men: 2.8	Women: ≤6.25=0 >6.25=1 Men: ≤2.8=0 >2.8=1

NND, New Nordic Diet.
